# Generalized Fracture Toughness and Compressive Strength of Sustainable Concrete Including Low Calcium Fly Ash

**DOI:** 10.3390/ma10121393

**Published:** 2017-12-06

**Authors:** Grzegorz Ludwik Golewski

**Affiliations:** Department of Structural Engineering, Faculty of Civil Engineering and Architecture, Lublin University of Technology, Nadbystrzycka 40 str., 20-618 Lublin, Poland; g.golewski@pollub.pl; Tel.: +48-81-538-4394

**Keywords:** sustainable concrete, low calcium fly ash, generalized fracture toughness, compressive strength, critical stress intensity factor, cracking, curing period, pozzolanic activity

## Abstract

The paper presents the results of tests on the effect of the low calcium fly ash (LCFA) addition, in the amounts of: 0% (LCFA-00), 20% (LCFA-20) and 30% (LCFA-30) by weight of cement, on fracture processes in structural concretes. In the course of the experiments, compressive strength of concrete and fracture toughness for: I (tensile), II (in-plane shear) and III (anti-plane shear) models of cracking were measured. The tests determined the effect of age of concretes modified with LCFA on the analyzed parameters. The experiments were carried out after: 3, 7, 28, 90, 180 and 365 days of curing. Fracture toughness of concretes was determined in terms of the critical stress intensity factors: $K_{Ic}^{S}$, $K_{IIc}$, $K_{IIIc}$ and then a generalized fracture toughness $K_{c}$ was specified. The obtained results are significant for the analysis of concrete structures subjected to complex loading. The properties of composites with the additive of LCFA depend on the age of the concrete tested. Mature concretes exhibit high fracture toughness at 20% additive of LCFA, while the additive of LCFA in the amount of 30% weight of cement has a beneficial effect on the parameters of concrete only after half a year of curing.

## 1. Introduction

The principles of sustained development and ecology are essential in the design of concrete compositions. However, it is well known that the basic binder in the composition of plain concrete, i.e., Ordinary Portland Cement (OPC), is not an environmentally friendly material. Cement manufacturing is one of the leading energy consuming and heavy pollutant processes which is accountable for CO_2_, NO_X_, SO_2_ emissions and some heavy metal discharge from the pre-calciner klin system. The world’s current Portland cement production contributes about 1.35 billion tons of carbon dioxide CO_2_ to the greenhouse gases in the earth’s atmosphere [1]. It should be noted that carbon dioxide has been identified as one of the main compounds affecting the stability of the earth’s climate, whereas the reduction of the total volume of greenhouse gases emitted into the atmosphere is considered a key mechanism to mitigate climate change.

It is estimated that the CO_2_ released during cement clinkering is around 0.7 to 0.9 tons per ton of OPC, meaning that the cement industry generates around 7% of total CO_2_ emissions worldwide [2]. The country that produces the largest amount of cement annually (57.5% of the world’s total in 2013) and thus is the world’s biggest emitter of CO_2_ is China [3]. Nevertheless in recent years, China has taken various long-term actions to significantly reduce the emissions of this harmful gas [3]. As far as Poland is concerned, the cement industry significantly reduced the emissions factor per ton of cement in the period of 1988–2008 by 28%. In 1988 the factor amounted to 0.879 tons CO_2_/tons of cement and 1.1 tons CO_2_/tons of clinker, while in 2008, respectively 0.631 tons CO_2_/tons of cement and 0.865 tons CO_2_/tons of clinker [4].

Therefore, concrete as the most popular building material has been included in the sustainable building program. It is connected with the fact that for many years the cement industry has been trying to follow the sustainable development strategy, whose main purpose is the reduction of environmental pollutions, primarily the reduction of CO_2_ emission as well as the usage of waste materials from other industries. The structures made of sustainable concrete are environmentally sustainable and are constructed in such a way that the total impact on the environment during their full life cycle, including service life, is reduced to a minimum. In this context, a responsible, sustainable construction should be energy efficient and made of environmentally friendly materials. Concrete used in such structures must meet the requirements for strength and durability and its components should be obtained, produced, and used in an environmentally friendly manner [5,6,7,8,9,10]. Furthermore, sustainable buildings are a necessary component of securing sustainability. According to [11] health, wellbeing, and effectiveness in those buildings is an important motivator for their occupants. Interesting proposals and suggestions for designing more sustainable concrete were proposed in [12]. Moreover, according to [13] the need for the utilization of waste materials is a demand of sustainable construction, whereas according to [14] along with technical and legal means, economic instruments are crucial components in the field of utilizing waste as substitutes of primary materials.

In this regard, for over several dozen years, the development of a new generation of concrete composites, i.e., environmentally friendly concrete composites, was followed by a significant increase of the production of concrete mixtures containing supplementary cementitious materials (SCMs), of different classes and types [15], often having pozzolanic properties, e.g., [16]. These are, among others: Silica fume (SF) [17], nano silica [18], ground granulated blast furnace slag (GGBFS) [19] and many types of fly ash (FA), including: Low calcium fly ash (LCFA) [17], high calcium fly ash (HCFA) [20], bottom ash [21], rice husk ash [22] and others [23]. 

Undoubtedly, one of the most commonly used mineral additives in the form of FA, is LCFA which is a porous waste by-product from coal combustion in thermal power plants. LCFA is formed by a multi-stage pyrolysis of hard coal, thoroughly described in [24].

As LCFA is a by-product obtained in the process of hard coal combustion, there are hundreds of millions tons of industrial waste produced annually across the World. LCFA is an abundant commodity in many countries using steam coal for power production. In some countries all the LCFA is utilized whereas in some others only a low percentage is used. LCFA utilization amounts to 39% in the USA and 47% in Europe, whereas the global average is estimated to be close to 25%, which is approximately 200 million tons per annum [25].

Nowadays, there is much research into the ability of LCFA to enhance: Construction, mining and terrain management. LCFA can be utilized in products such as: Building materials, zeolite synthesis, wood substitute products, soil stabilization, road base/embankments and ground consolidation, land reclamation, and soil amendments in agricultural applications, e.g., [26]. A comprehensive review of the applications of LCFA was presented in [27]. However, the main mode of utilization is in the civil engineering industries as cement or clinker additive or for concrete production. According to [28], the major worldwide use of LCFA is in concrete, which exceeds any other single application. Additionally, the use of LCFA in concrete reduces the greenhouse emissions equivalent to the average annual emissions from 2.5 million cars [29]. Hence, a significant reduction in greenhouse emissions can be achieved by increasing the utilization of LCFA in concrete.

The issues connected with the substitution of cement binders with LCFA are important not only from the scientific point of view, but also in terms of applicability, from the economic point of view. Although, the governments of many countries had a long discussion on the perspectives and strategies of the implementation of diversified energy systems, the prognosis concerning the energy production from coal as the main source stays stable. On the basis of [29], coal usage is expected to increase 3.4% over the next two decades despite the retirement of many coal plants.

Concrete composites with the addition of LCFA can certainly be included in the sustainable and environmentally friendly concrete category. Nowadays, in the cement and concrete industry, LCFA is used for production both in plain concretes as well as in novel composites, e.g., as an addition to nano concrete [30]. As the concretes with these additives are frequently used as a traditional or modern useful building materials, they require a thorough examination.

## 2. Discussion of Scientific Objectives

The overall objective of the conducted scientific analyses was a description of fracture processes in concretes with the LCFA additive. Concretes with these additives have been used in the industry for approximately 80 years, and in the past two decades their use in the composites with cement matrix has significantly increased. Therefore, it is obvious that in the course of such a long period of time the effect of the LCFA additive on the basic physical and mechanical characteristics of concrete could be thoroughly explored. However, a closer look at the literature devoted to this area of science suffices to reveal a scarcity of complete data related to the analysis of initiation and propagation of microcracks and cracks developing in result of the impact of complex stress states on concrete.

LCFA is a pozzolanic material that demonstrates binding capacity through active LCFA ingredients (mainly SiO_2_ and Al_2_O_3_) calcium hydroxide. These reactions lead to the increase of C-S-H phase in concrete, which has a significant effect on the properties of concrete. The introduction of LCFA to the composition of cement causes changes in the phase composition and the microstructure of cement paste which is important for the development of mechanical parameters of concrete. Typically, composites with greater diversification of phases are characterized by higher sensitivity to the formation of local stress concentrations, which may imply the presence of damages and microracks in these locations. Such defects occur mainly in the areas of adjoining structures; under the influence of increasing external loads, the development, accumulation and, consequently, the premature material destruction occurs.

In concrete, cracks may arise from shrinkage, freeze-thawing and (or) structural stresses, amongst others [31]. Full knowledge of cracking processes in concrete structures is very important because the properties of concrete materials, including their durability, mainly depend on the structural factors and the interaction between micro- and macrostructure of the material. Cracks and losses are the two basic defects of the concrete structure, which may reduce the load-bearing capacity, leak tight integrity and stiffness of elements and structures which, in extreme cases, may lead to failures and even building catastrophes. According to [31], cracking is a major concern in building applications, whereas according to [32], cracking is one of the primary causes of deterioration in concrete composites and concrete structures.

In order to carry out an in-depth analysis of cracking processes occurring in concrete structures, i.e., crack initiation and its growth, the engineers, most frequently, use non-destructive methods, e.g., [33]. Numerical simulations are also an effective and practical alternative to the experimental studies investigating fracture in heterogeneous materials, such as concrete [34,35]. However, for engineers designing concrete mixtures, it is also important to be familiar with the processes of crack initiation and of propagation of structural materials, especially those that are characterized by high brittleness. Such knowledge would allow them to enhance the quality of concrete destined for structures, to estimate defects and determine their causes, and, finally, to obtain composites with the highest levels of durability and reliability.

The reduction of concrete strength results from the initial structural defects existing inside material [36], mainly in the Interfacial Transition Zone (ITZ) between coarse aggregate and cement paste [37]. Therefore, the cracks in structural elements usually start to initiate in the mixed mode loading, whereas failure of concretes is a multi-stage process that is conditioned by level and type of the applied external loads as well as by the internal structure of composite [38]. This is connected with the fact that fracture is an important feature in concrete at all scales [39], whereas cracks and notches in engineering components and structures are often subjected to complex loading conditions which generate different mixtures of normal and shear stresses ahead of the crack tip [40]. Moreover, the damage of concrete subjected to complex loading induces strong anisotropy due to its highly heterogeneous nature and geometrically anisotropic characteristic of microcracks. Thus the 3-D fracture process is generally complicated, i.e., an experimental estimation of all fracture mechanics parameters is extremely difficult and can be carried out for three separate fracture modes: I, II, and III.

Seeing high degree of heterogeneity of concrete composites one can state that, even in the case of the ideal external load, local interactions between the grains and pores cause the occurrence of all three models of cracking.

**Figure 1:**
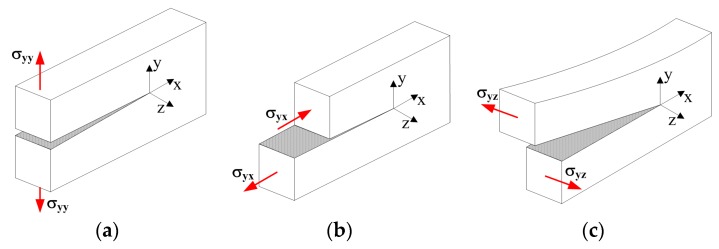
Different fracture modes: (**a**) mode I; (**b**) mode II; (**c**) mode III. $\sigma_{yy}$, $\sigma_{yx}$, $\sigma_{yz}$—stresses causing growth of initial crack.

On the basis of the above results, it can be concluded that a thorough analysis of crack initiation and propagation requires the knowledge of all fracture mechanics parameters for modes: I, II and III, i.e., $K_{\mathrm{Ic}}$, $K_{IIc}$, $K_{IIIc}$, e.g., [41,42,43,44]. Figure 1 defines the three modes of loading, i.e.,

- mode I, opening or tensile mode,
- mode II, sliding or in-plane shear (pure shear) mode,
- mode III, tearing or anti-plane shear mode.

The tests of fracture toughness in complex stress states are associated with the description of the present critical state in the element, which can be written as the following Relationship (1):

(1) $$f\left(K_{Ic},K_{IIc},K_{IIIc}\right)=\;f_{c}$$

With the known values of critical stress intensity factors: $K_{Ic}^{S}$, $K_{IIc}$, $K_{IIIc}$, it is possible to designate a generalized fracture toughness of the material—$K_{c}$ from the Equation (2) [45]. The reason why the stress intensity factor is useful is that fracture (sudden crack growth to failure) occurs when K exceeds a certain critical value $K_{c}$ called exactly the fracture toughness.

(2) $$K_{c}^{2}=K_{Ic}^{2}+K_{IIc}^{2}+\frac{K_{IIIc}^{2}}{1-\nu }$$

where: v is Poisson’s ratio.

Unfortunately, up till now, the question of LCFA influence on fracture toughness of concrete composites was not investigated thoroughly. In the assessment of fracture toughness of concretes containing LCFA the available results of experiments concern mainly the first mode fracture and seldom the second one. Fracture toughness under mode I—$K_{Ic}$ and mode II—$K_{IIc}$ for plain concretes containing LCFA additives are the topics of several articles [46,47,48,49,50], while only three papers presents the fracture toughness for the mode III—$K_{IIIc}$ [51,52,53] and work of fracture—$W_{f}$ [38]. The comprehensive study [38], devoted to cracking processes of concrete specimens containing LCFA under torsional loading, examined the effect of the LCFA additive on the $W_{f}$. Furthermore, the paper [38], assessed the values of critical torsion moment $T_{c}$ and dissipated energy $\Delta_{W}$ at the fracture initiation point.

An additional problem is the fact that all previous papers usually refer to the study of the fracture toughness of concrete containing significantly different amounts of LCFA (from 20 to 55%) regularly after 28 (and optionally after 56) days of their curing.

This paper provides new testing methods and novel experimental results concerning generalized fracture toughness in the investigated concretes. The studies present original research techniques, e.g., in order to determine the fracture toughness $K_{IIIc}$, a special device has been manufactured and patented.

In particular, the paper contains a comparative analysis of the generalized fracture toughness $K_{c}$ of plain concretes and concretes modified with the LCFA additive. The reason to initiate the work on fracture processes analysis in concretes with the LCFA additive lies in the fact that the strength of such materials increases in the process of curing [54]. Due to the slow course of the pozzolanic reaction, which directly influences the mechanical properties of composites of this type, the strength increases slowly at the initial stage of hardening [55]. However, when curing over longer time, the cement with LCFA achieves values exceeding the compressive strength of Portland cement of the same strength class.

So far, the analysis of the effect of curing time in low- and high volume LCFA concrete aimed at the evaluation of important properties, e.g., Mechanical properties, corrosion resistance, water absorption, water permeability, and frost resistance or microstructure and crack propagation. A list of papers describing these studies are summarized in Table 1.

In the present study—having in mind the reason of working on this subject—we examine changes in concrete compressive strength, $f_{cm}$, and in fracture toughness of concretes at early ages and in a period exceeding 28 days. As concrete structures are usually subjected to loads which cause complex stress states in the cross-sections of elements, the analysis provides the characteristics of fracture toughness in the specimens corresponding to the development of cracks in the material under: I, II and III mode fracture. The results obtained give several practical tips and recommendations concerning the use of composites with the LCFA additive in the concrete industry.

All experiments were planned for two compositions of concrete mixture, with varying percentage of LCFA additive often used in the cement industry. The choice of the target range was determined by the fact that in the tests (their results were presented in paper [48], a pilot study), fracture toughness under II mode fracture was analyzed in concretes with the LCFA additive in the amounts of: 10%, 20% and 30% of weight of cement. On their basis, it was found that 10% LCFA additive has a minor impact on the value of parameter $K_{IIc}$ causing its increase by only 1% while 20% and 30% additive of this waste significantly changes the fracture toughness.

Therefore, all tests were conducted for concretes modified with the LCFA additive in the amount of 20% (LCFA-20) and 30% (LCFA-30) of the weight of cement. The results of the experiments were compared to the values obtained for the reference concrete (LCFA-00), which was a composite made with the use of cement CEM I. For each of the three composites, all experimental tests were conducted in six time periods, with the age of concrete: 3, 7, 28, 90, 180 and 365 days.

An advanced diagnostic equipment was used during the experiments. For the evaluation of fracture toughness, three separate test stands were organized (see Section 3.6). On the basis of the planned scope of work, associated with the realization of the academic achievement, the complete repository of the detailed academic objectives is as follows:

1. In the range of LCFA testing, the scientific objectives were:
  - analysis of physicochemical properties of LCFA,
  - evaluation of pozzolanic activity of LCFA.
2. In the range of fracture toughness, the scientific objectives were:
  - experimental determination of the impact of LCFA additive on the critical stress intensity factor of concrete under: I, II and III mode fracture, between the third and 365th day of curing,
  - calculation of particular concretes with generalized fracture toughness $K_{c}$.

## 3. Experimental Section

### 3.1. Materials

The concrete mixtures were prepared at the laboratory of the Faculty of Civil Engineering and Architecture, Lublin University of Technology (Lublin, Poland). The following materials were used: OPC, LCFA, sand, gravel, plasticizer and water.

In this research, both OPC and LCFA were used as a binder, OPC having the compressive strength equal to 23.3 MPa on the second day and 50.0 MPa after 28 days of curing. The LCFA used, in different percentage, to replace cement is a product of energetic combustion of hard coal in the local thermal-electric power station. Bulk density of both materials was determined by pycnometric method, whereas the specific surface area by Blaine method. The specific density of OPC and LCFA used was 3.11 and 2.14 kg/m^3^, respectively. The fineness specific surface of OPC and LCFA used was 328 and 364 m^2^/kg, respectively. The volume change of OPC used was 0.56 mm with an initial and final setting time of 207 and 298 min, respectively.

**Figure 2:**
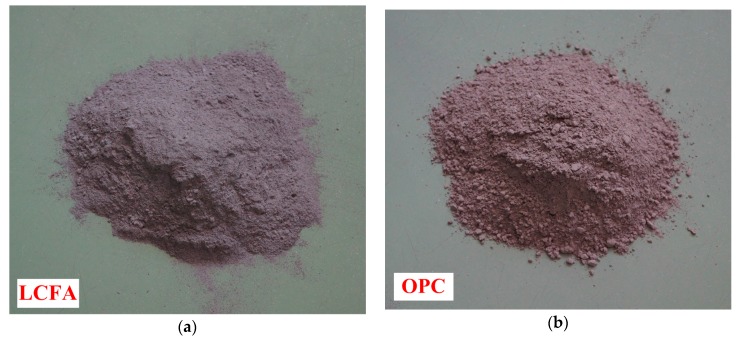
Apperance of the binders: (**a**) LCFA; (**b**) Ordinary Portland Cement (OPC).

The characteristics of typical grains, microstructures, and the dominant morphologies of OPC and LCFA particles can be found in [38,49,51], whereas Figure 2 shows the appearance of OPC and LCFA. In the macroscopic appearance (in real) the OPC grains are light grey, whereas the LCFA grains are dark grey.

The mineralogical composition of OPC was analyzed by the Bogue method. These studies showed that, in OPC tested, there are (in %) the following phases: C_3_S—60.69, C_2_S—15.82, C_3_A—9.24, C_4_AF—7.28, CaSO_4_ (gypsum)—5.10. X-ray diffraction (XRD) analysis allowed for the identification of the crystalline phases of LCFA [48] and showed that, besides glass, there are 4 major crystalline components in the phase composition of LCFA, namely: Quartz (SiO_2_), mullite (Al_6_Si_2_O_13_), magnetite (Fe_3_O_4_), and hematite (Fe_2_O_3_) [48].

We evaluated the chemical composition of OPC and of LCFA by X-ray fluorescence (XRF). Table 2 shows the chemical composition of the OPC and the LCFA used in this study. LCFA is a class F with 85.09% of SiO_2_ + Al_2_O_3_ + Fe_2_O_3_, 0.65% of SO_3_ and 3.2% of Loss of Ignition (LOI), meeting the requirement of ASTM C618 standard.

Three aspects of natural radioactivity of LCFA, and of concrete containing it, were considered: Concentration of radioactive elements (potassium, radium and thorium), radioactivity coefficients $f_{1}$ and $f_{2}$, and the radiation dose [67]. The study of natural radioactivity of LCFA shows that these materials are not radiologically hazardous. The levels of concentration of radioactive elements, radioactivity coefficient, and the radiation dose were contained within the acceptable limits [67].

**Figure 3:**
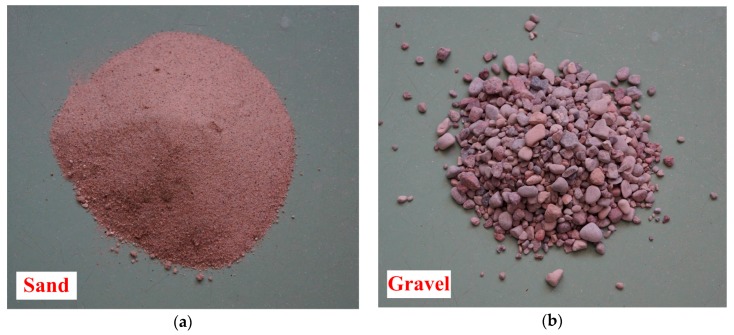
Aggregates used in the studies: (**a**) Sand; (**b**) gravel.

Concrete mixes were designed using natural pit sand 0–2 mm (Figure 3a), natural gravel 2–8 mm (Figure 3b), with water-binder *w*/*b* ratio 0.4.

A calcium lignosulfonate-based plasticizer used in this study had density of 1.16 g/cm^3^ and the dosing range of 0.1–1.0% of mass of cement. The plasticizer is used in an amount of 0.6% of mass of the binder.

### 3.2. Preparation and Casting of Test Specimens

The composition of prepared concrete mixes is shown in Table 3. The cast specimens were covered with a polyurethane sheet and a damped cloth and placed in 20 ± 2 °C chamber. After 2 days all specimens were demoulded and kept for the first 14 days in a chamber with a moisture-saturated atmosphere. After: 28, 90, 180 and 365 days the specimens were cured in laboratory conditions (at 20 ± 2 °C), the specimens tested after 3 and 7 days were taken out of the water at least a few hours before the study. After a suitable period of curing time we carried out the compressive strength tests and fracture toughness tests.

### 3.3. Specimens Used in the Studies

Assortment of specimens for compressive strength tests and fracture toughness tests of concrete, for each of the mixtures in all time periods, was as follows:

- 6 cubic specimens (150 mm) for testing the compressive strength—$f_{cm}$,
- 6 beams (80 × 150 × 700 mm) with one initial crack for testing fracture toughness under mode I fracture—$K_{Ic}^{S}$,
- 6 cubic specimens (150 mm) with two initial cracks for testing fracture toughness under mode II fracture—$K_{IIc}$,
- 6 cylindrical specimens with a diameter of 150 mm and a height of 300 mm having an initial circumferential notch, for testing fracture toughness under mode III fracture—$K_{IIIc}$.

### 3.4. Pozzolanic Activity of the LCFA

The applicability of the LCFA depends mainly upon its pozzolanic activity. To assess the pozzolanic activity of the used LCFA we selected a physical method, according to the EN 450-1 standard. EN 450-1 defines as a standard pozzolanic activity index of the LCFA the Strength Activity Index which is the ratio (in %) of the compressive strength of mortar containing 75 wt % of cement CEM I 42.5R, 25 wt % of the LCFA and cement mortar without addition. According to EN 450-1 standard the pozzolanic activity index of the LCFA is determined after 28 and 90 days of hydration. The compressive strength of cement is measured according to the procedure described in EN 196-1 standard, using six 40 × 40 × 160 mm^3^ prisms of the mortar. The water to solid ratio in mortars is 0.5. According to the EN 450-1 standard, the acceptable level of pozzolanic activity is attained when the 28 day compressive strength of the LCFA and cement mortar constitutes 75% of the value for the control sample and after 90 days, 85% respectively.

Due to that compressive strength tests and fracture toughness tests were conducted in 6 time periods between 3 and 365 days, pozzolanic activity index was also determined at the same time intervals, i.e., after: 3, 7, 28, 90, 180 and 365 days.

### 3.5. Compressive Strength Test

We performed compressive tests (Walter + Bai ag, type NS19/PA1; Löhningen, Switzerland) for uniaxial compression strengths with maximum load of 3000 kN while controlling the loading rate between 0.5 MPa/s and 0.8 MPa/s. The compressive strengths were tested with application of cubic specimens (see Section 3.3) according to the standards of series EN 12390.

### 3.6. Fracture Toughness Tests

#### 3.6.1. Mode I Loading

**Figure 4:**
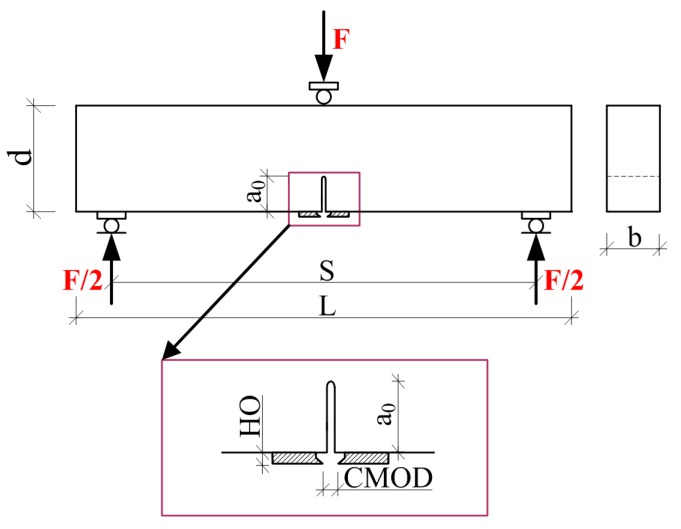
Schematic drawings of the specimen used in the fracture toughness examination according to mode I: HO—clamp gauge holder thickness, CMOD—crack mouth opening displacement.

The testing of mode I fracture toughness was performed in accordance with the draft guidelines of RILEM recommendations [68]: *d* = 150 mm, *b* = 80 mm, *L* = 700 mm, *S* = 600 mm, *a*_0_ = 50 mm (Figure 4). 

The fracture toughness of the composites with the LCFA addition was determined on the bases of the experimental results of critical stress intensity factor $K_{Ic}^{S}$, e.g., [69,70].

**Figure 5:**
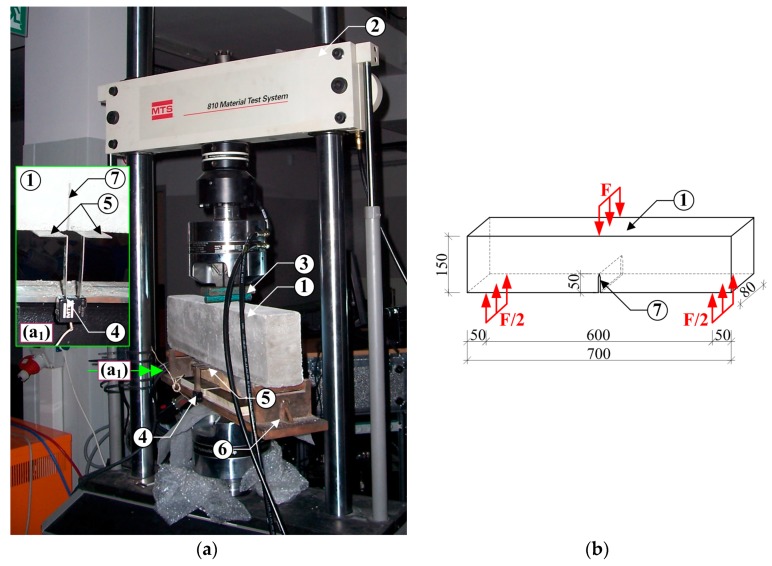
View of experimental stand for testing according to mode I fracture: (**a**) specimen during testing with the detail (a_1_) the position of the extensometer in the grips; (**b**) specimen with dimensions and loading conditions; 1—specimen, 2—MTS 810 press, 3—system applying force onto the specimen, 4—axial clip gage extensometer, 5—clamping test grips, 6—supporting system, 7—initial crack (dimensions in mm).

To assess the fracture toughness of concrete, beams with one initial, centrally situated crack, were used (see Section 3.3). The beams were subjected to 3-point bending test [69]. They were made in demountable bolted wooden forms. The assumed size of the initial crack in the beams was achieved by actually concreting flat steel plates, 3 mm thick. The tests setup apparatus is displayed in Figure 5. Figure 5 shows the whole experimental stand with details (Figure 5a) and the specimen dimensions with loading conditions (Figure 5b).

All results necessary to determine the critical stress intensity factors for concretes, were obtained with MTS 810 testing machine. The width of the initial crack opening during the tests was measured by a crack opening sensor, that is the MTS clip gage axial extensometer, which was placed on the clamping test grips (Figure 5). 

**Figure 6:**
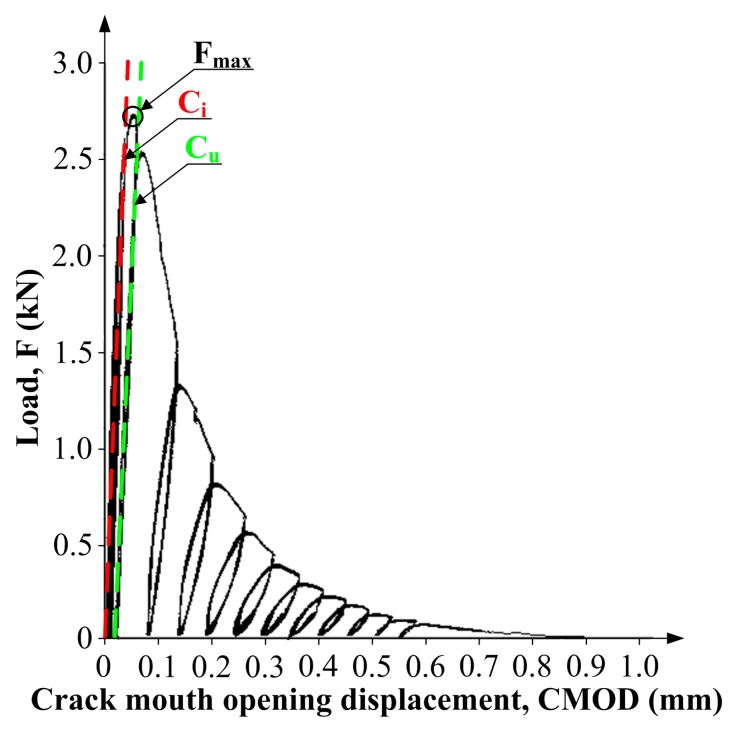
An example graph of CMOD-load relationship obtained in the tests at mode I fracture.

The specimens placed in the experimental stand were subjected to cyclic loading process performed quasi statically. Loading rate was selected so that the maximum load— $F_{max}$ (Figure 6) was reached in approximately 5 min. The applied load was reduced at approximately 95% post-peak load. Having the load reduced to 0 kN, the test specimen was reloaded. After that the cycles were repeated (6 to 8 times) until the beams were broken into 2 parts. The whole cyclic deformation processes were described by the following curves:

- Load (*F*)—Crack mouth opening displacement (CMOD),
- Load—Displacement (*f*).

An example plot of CMOD vs. load increments is shown in Figure 6. Based on the plots obtained for each test specimen, the following quantities were determined: Young’s modulus—*E*; critical effective crack length—$a_{c}$; critical stress intensity factor—$K_{Ic}^{S}$ and critical crack tip opening displacement—$CTOD_{c}$ [68].

In the graph illustrating a relationship *F-CMOD*, a tangent in the first phase is highlighted in red and in the second phase, in green. With the given slope of the curve *F-CMOD* in initial cycles of loading a specimen, it was possible to determine the critical stress intensity factor $K_{Ic}^{S}$.

The critical stress intensity factor $K_{Ic}^{S}$ was calculated from the Equation (3) [68]:

(3) $$K_{Ic}^{S}=3\left(F_{max}+0.5W\right)\frac{S{\left(\pi a_{c}\right)}^{1/2}F\left(\alpha \right)}{2W^{2}b},$$

in which:

$$F\left(\alpha \right)=\;\frac{1.99-\alpha \left(1-\alpha \right)\left(2.15-3.93\alpha +2.7\alpha^{2}\right)}{\sqrt{\pi^{1/2}\left(1+2\alpha \right){\left(1-\alpha \right)}^{3/2}}},$$

where: $\alpha =\;\frac{a_{c}}{d};\;F_{max}$, the masured maximum load; *W* $=\;\frac{W_{0}S}{L};$ $W_{0},$ self-weight of the beam *d*, *b*, *S*, *L*—according to Figure 4.

#### 3.6.2. Mode II Loading

**Figure 7:**
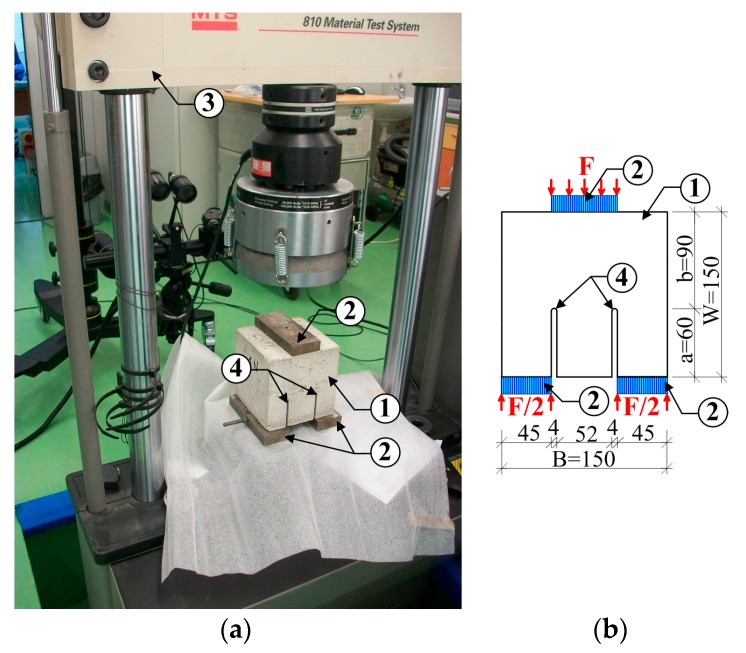
View of experimental stand for testing according to mode II fracture: (**a**) specimen during testing, (**b**) specimen with dimensions and loading conditions; 1—specimen, 2—stiff plate, 3—MTS 810 press, 4—initial crack (dimensions in mm).

Tests method to evaluate concrete fracture toughness $K_{IIc}$ is loading specimens according to the mode II fracture. In the experiments cubic specimens with two fictitious crack (type of compact shear) were used (see Section 3.3). The target crack sizes were obtained by embedding two 4 mm steel sharpened flat plates in concrete cubes being formed. The details of specimen preparation were described in [48], whereas Figure 7 shows the whole experimental stand (Figure 7a) and specimen dimensions with loading conditions (Figure 7b).

**Figure 8:**
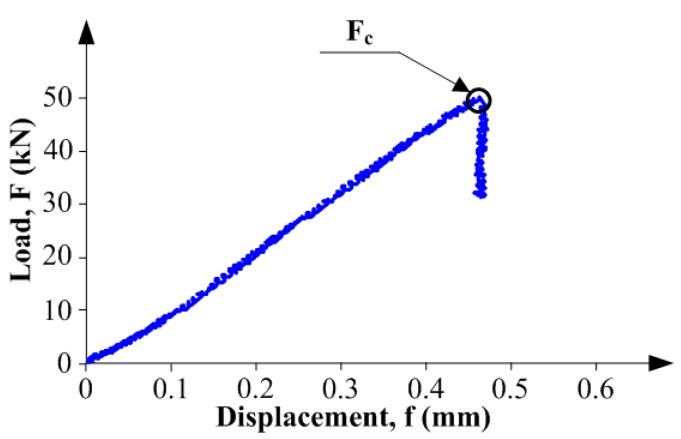
An example graph of load-displacement relationship obtained in the tests at mode II fracture.

The tests were performed with the use of the MTS 810 hydraulic press (Figure 7a), like in the studies under mode I fracture. During the tests, the specimens were loaded with a steadily increasing force. The force increase was regulated on the basis of the speed of the press head displacement in the function of time. The displacement value was assumed at the level of 0.25 mm/min. On the basis of data recorded during the tests, there were plotted the graphs of:

- Load (*F*)—Time (*t*),
- Load—Displacement (*f*),

and the values of fracture toughness were calculated, according to Equation (4), proposed by Watkins [71].

(4) $$K_{IIc}=\frac{5.11F_{c}}{2Bb}\sqrt{\pi a,}$$

where: $F_{c}$, value of critical force corresponding to beginning of the crack growth, this stage is identified on the failure diagrams as a small collapse or extremum of curve (Figure 8); *B*, *b*, *a*—according to Figure 7.

#### 3.6.3. Mode III Loading

**Figure 9:**
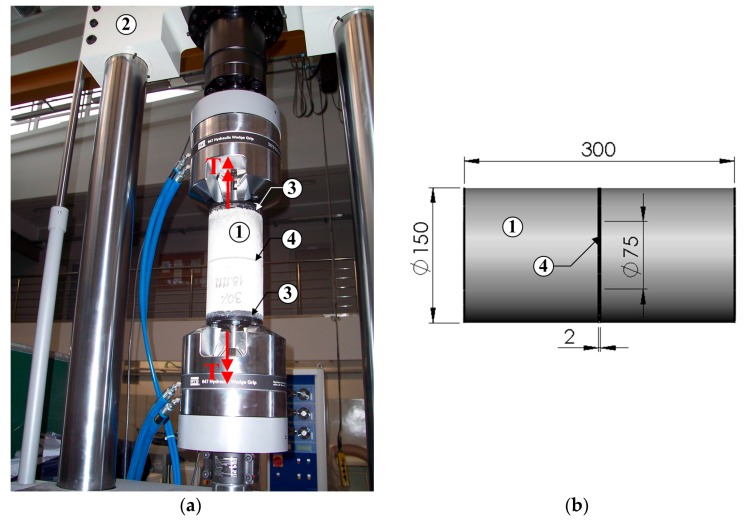
View of experimental stand for testing according to mode III fracture with loading conditions: (**a**) the device during testing, (**b**) specimen with dimensions; 1—specimen, 2—MTS 809 press, 3—steel elements, 4—initial crack, *T*—torque (dimensions in mm).

For tests being in accordance with III model of cracking (anti-plane shear), a special innovative device was designed and manufactured (Figure 9). This device has also been patented [72].

The device for the fracture toughness test at the mode III fracture consisted of the: cylindrical specimens with a diameter of 150 mm and a height of 300 mm having an initial circumferential notch of 2 mm thickness (Figure 9b), steel plates, and screws with washers securing the specimen in the press holders. Steel elements in the device were used for precise mounting of the specimen in the holders of the testing machine. Initial notch depth was equal to ¼ of the diameter of the cylinders. Initial notches in the specimens were created during their formation with two semi-circular steel inserts placed in the half-height of specially prepared cylindrical forms.

To mount the specimens in the grips of the torsional testing machine, two types of circular steel plates with drilled holes were designed and manufactured. During the forming process, the bottom plates with 15 mm thickness were anchored in the specimens at their top and the bottom through 6 bolts M12/65. Then, top plates with 10 mm thickness were bolted to these plates through 6 bolts M12/20. These plates hold the specimen directly in the grips of the torsion testing machine from the top and bottom through M28/70 bolts. More details of the device preparation were described in [51], whereas the full device for testing the $K_{IIIc}$ is shown in Figure 9.

The specimens were tested on the axial tension-torsion testing machine MTS 809 in accordance with the load diagram, also shown in Figure 9a. The shear process of specimens was controlled by rotation angle ω assuming a small increase equal to 0.5°/min. On the basis of the data recorded during the tests, the graphs of:

- Angle of rotation (ω)—Time (*t*),
- Torque (*T*)—Time,
- Torque—Angle of rotation,

were plotted and the values of fracture toughness were calculated.

**Figure 10:**
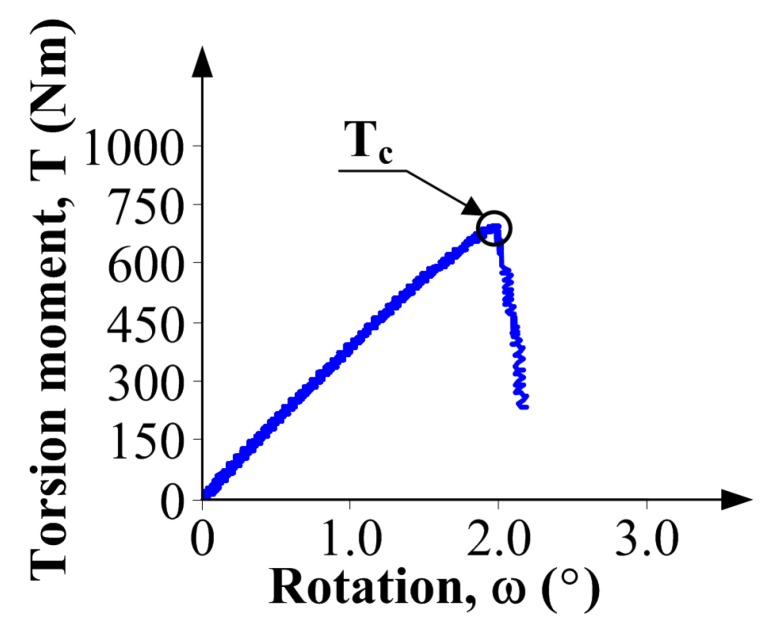
An example graph of torsion moment-rotation relationship obtained in the tests at mode III fracture.

Critical values of a torque ($T_{c}$) and the rotation angle $\omega_{c}$ which correspond to the failure of the specimens were reached after a few minutes. The failure, i.e., the moment when the specimen no longer carry the torque $T_{c}$, is usually characterized by a sudden collapse on the graph T(ω) (Figure 10).

On the basis of the experimental results, fracture toughness $K_{IIIc}$ can be calculated, according to the Equation (5), proposed by Miannay [73].

(5) $$K_{IIIc}=\frac{\tau_{c}\sqrt{2\pi r}}{\cos\left(\frac{\omega_{c}}{2}\right)},$$

where: $\tau_{c}$, maximum stress corresponding to $T_{c}$ (Figure 10); *r*, radius of the specimen (Figure 9); $\omega_{c}$, critical rotation angle corresponding to $T_{c}$ (Figure 10).

#### 3.6.4. Determination of Generalized Fracture Toughness

With the known values of $K_{Ic}^{S}$, $K_{\mathrm{IIc}}$ and $K_{\mathrm{IIIc}}$, it was possible to designate a generalized fracture toughness of the material—$K_{c}$ from the Equation (2), in all time periods.

## 4. Test Results

### 4.1. Pozzolanic Activity

**Figure 11:**
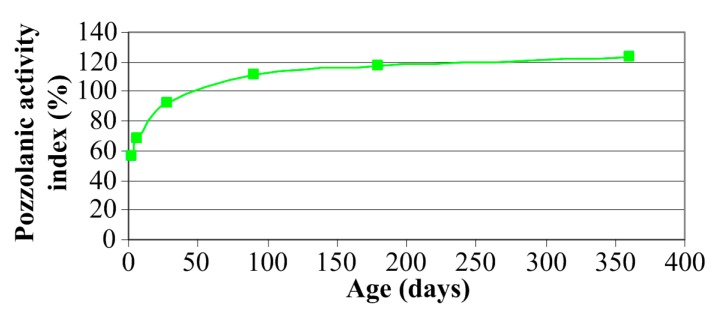
Pozzolanic activity index vs. age of mortar.

Figure 11 shows how the growth rate of the Strength Activity Index looked. A line connecting points on the graph (Figure 11) shows a gradual, nonlinear increase of the activity of the LCFA between the 3rd and 365th day of curing of samples. After three days, the Strength Activity Index is very low, which is certainly due to the slow growth of the reaction products after such a short period of curing. According to the results of previous studies, e.g., [65], after one day LCFA is still inactive and pozzolanic reaction in this material begins in one to three days.

Strength Activity Index (Figure 11) indicates that the initial effect of the pozzolanic reaction (consisting in strengthening the matrix) in the composites which include the addition of LCFA, can be observed after 7 days of curing. This is also confirmed by other studies, e.g., [56]. After this time, a significant increase occurs in the activity of the material of more than 20%. In the period between the seventh and the 28th days there was already visible an intense increase in the strength of mortars with LCFA and the activity index (when compared to the value of weekly increases by almost 35%). A significant increase in the analyzed parameter is visible even between the 28th and the 90th days of curing of the samples. In a further period of time, between three to six months, and after a year, there has been a slight increase of the pozzolanic activity of LCFA (by a few percent). It can be concluded that after six months, the stabilization of the ongoing reactions is already achieved. Nevertheless, the reactions still take place even after 365 days [56].

As a result of the study it can be concluded that the pozzolanic activity of the LCFA meets the requirements of EN 450-1 standard. Strength Activity Indices were at 28 days, 92.13%, and after 90 days, 111.84%, which means that they exceed significantly the minimum values specified in the requirements of the EN-450-1 standard.

### 4.2. Compressive Strength

**Figure 12:**
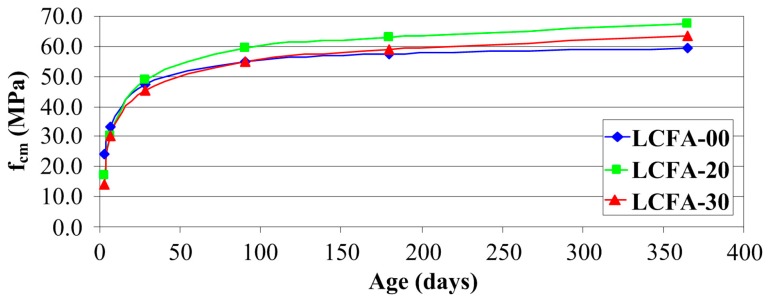
Compressive strengths of analyzed concretes as a function of curing time.

The compressive strength data (average values of six specimens, see Section 3.3) for: 3, 7, 28, 90, 180 and 365 days of age of sustainable concrete made with and without LCFA are plotted in Figure 12. As expected, $f_{cm}$ increased with age in all the concrete specimens. Figure 12 shows the variation of $f_{cm}$ with LCFA percentage at different ages.

As expected, the early strength gain of the control mixtures without LCFA was superior to that of the LCFA mixture, a decrease in compressive strength was observed as the percentage of LCFA replacement increased. For example, at three days of curing the compressive strength at 20% and 30% replacement levels were 16.95 and 14.23 MPa, respectively, whereas at seven days of curing the $f_{cm}$ at 20% and 30% replacement levels were 30.12 and 30.06 MPa, respectively. According to Figure 12, value $f_{\mathrm{cm}}$ after 72 h of curing was almost eight and exactly 10 MPa higher in concrete without the LCFA additive in comparison to LCFA-20 and LCFA-30, respectively. After a week, the differences between the reference and modified concretes were only three MPa. According to [49], presenting a more detailed analysis of an early stage changes of strength parameters in concretes with the LCFA additive at a early age is presented, clear disproportions in the obtained results occur within 14 days of curing. After three weeks, 20% LCFA additive strengthens the structure of concrete composites to the extent that, in comparison to LCFA-00, the values of $f_{\mathrm{cm}}$ in this type of concrete are slightly higher. Also, after 28 days and in subsequent time periods, LCFA-20 had, by far, the highest strength, which was probably due to the sharp increase of pozzolanic reaction products after a longer time of curing. Concrete with a larger amount of LCFA was characterized by the lowest strength in the period up to three months. After a half year of curing, the value of $f_{\mathrm{cm}}$ for this composite was higher in comparison to concrete without the additives, however, still lower than for LCFA-20. Further increase of strength of LCFA-30 caused that, after a year, the strength of this material was 4 MPa higher $f_{\mathrm{cm}}$ in comparison to LCFA-00 and lower by the same value in comparison to LCFA-20. After 365 days of curing, the highest $f_{cm}$ was noted in the 20% LCFA cement concrete specimen (67.29 MPa) followed by those prepared with 30% LCFA (63.27 MPa) and finally the control concrete (59.25 MPa).

**Figure 13:**
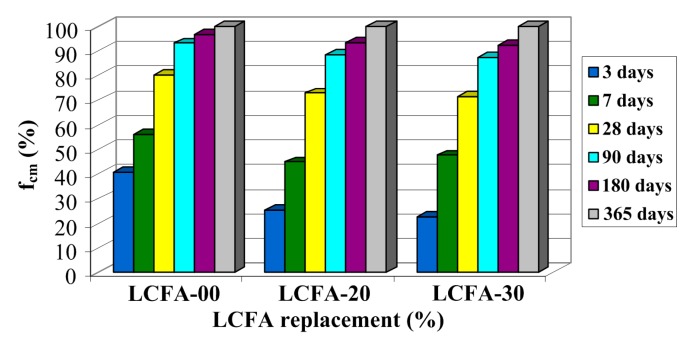
Relative changes of compressive strengths over time.

The tests we have conducted have shown that compressive strength of concrete was increasing with time. The growth dynamics of this parameter, however, differed significantly in particular types of the analyzed composites. This can be easily observed by comparing the relative changes of compressive strength over time, which is shown in Figure 13.

After the first measurement, i.e., after three days, concrete FA-00 had more than 40% of the annual compressive strength, whereas in concretes with LCFA additives, this strength did not even had 30% of the final strength. After a week, concrete, without the LCFA additive, was characterized by a higher relative strength, however, in the analysis of the obtained values, a clearer dynamics of strength increase in composites with LCFA was observed. Although the reference concrete had already more than 50% of its 365 day strength, which was a result 10% better in relation to concretes with additives, a greater increase of $f_{\mathrm{cm}}$ was observed in concretes with LCFA between the third and seventh day of curing. Also, between the seventh and 28th of curing, concretes with additives are characterized by significant increments of $f_{\mathrm{cm}}$ (amounting to: LCFA-20—50%, LCFA-30—62%), which correlates with the activity of pozzolanic LCFA obtained with the use of physical method in accordance with EN 450-1 (Figure 11). Clear growth trends of compressive strength, in modified composites, was observed even after 90 days. Both 20% and 30% LCFA additives caused the increase of $f_{\mathrm{cm}}$ within two months by more than 20%, which was a result 6% better in relation to LFA-00. After half a year in all types of composites, the increase of compressive strength was small and was 6% for modified concretes and only 3% in the reference concrete. A similar trend was also observed in relation to one year concretes where the strength increase in concretes with LCFA was 7% and was higher by half in comparison to the results obtained for LCFA-00.

### 4.3. Generalized Fracture Toughness

**Figure 14:**
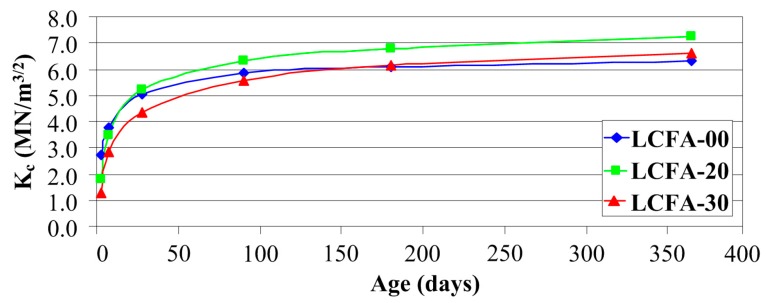
Generalized fracture toughness of analyzed concretes as a function of curing time.

The generalized fracture toughness data (average values) for: 3, 7, 28, 90, 180 and 365 days of age of sustainable concrete are plotted in Figure 14.

Figure 14 shows the variation of $K_{c}$ with LCFA percentages and in this figure it can be seen that the generalized fracture toughness increased with age in all concrete specimens. The effect of LCFA on $K_{c}$ parameter was similar to their effect on $f_{cm}$. Similarly to the strength tests results, values $K_{c}$, in the initial stages of curing of concretes, were also very low. This could be clearly seen in composites with the LCFA additive where after three days, generalized fracture toughness was 34% and 52% lower for LCFA-20 and LCFA-30, respectively, in comparison to the value obtained for LCFA-00. In the subsequent time periods, for mature concretes, a sharp increase of generalized fracture toughness occurred in concrete with the 20% LCFA additive, for which values of $K_{c}$ clearly exceeded the results obtained for two other materials. Larger amount of micro-filler in the structure of concrete made LCFA-30 reach the lowest generalized fracture toughness in the first three months. However, in mature concretes the highest generalized fracture toughness had LCFA-20 and LCFA-30 after 180 and 365 days.

**Figure 15:**
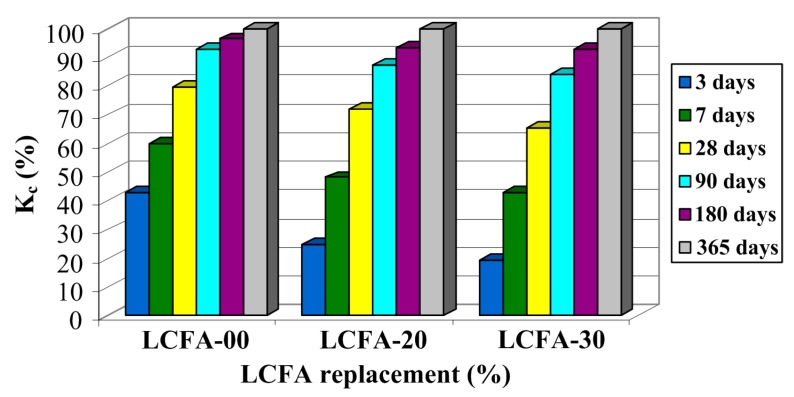
Relative changes of generalized fracture toughness over time.

The graph of relative changes $K_{c}$ shown in Figure 15 is remarkably similar to the results presented for $f_{cm}$ in Figure 13. A clear graphical correlation between the graphs shown in Figure 13 and Figure 15 can be found. A similar correlation between the results of both analyses is also shown in Figure 12 and Figure 14 which present, in the analyzed concrete, a similar rate of change in compressive strength and generalized fracture toughness over time. This means that the results of the generalized fracture toughness and the compressive strength are convergent qualitatively.

After comparison the relative changes of generalized fracture toughness in time (Figure 15), one can conclude that the rate of increase of this parameter largely depends on the type of the analyzed composite. As concrete LCFA-00 reached nearly 43% of the final value of $K_{c}$ after three days, the subsequent increase of fracture toughness for this material was already quite stable. The three-day values of $K_{c}$ with respect to 365-day values were 25% and 19% for LCFA-20 and LCFA-30, respectively. Also, after a week, large disproportions were noticed in the obtained results. The value of $K_{c}$ in concrete LCFA-00 accounted for almost 60% of the annual result. At the same time, in concretes with LCFA, generalized fracture toughness was much less than 50% $K_{c}$ obtained for these concretes after 365 days. Sharp increase of $K_{c}$ for concretes with the LCFA additive was observed between seventh and 90th day of curing. During this time, generalized fracture toughness for these materials increased by 55% for LCFA-20 and 51% for LCFA-30. In all of the analyzed concretes, values $K_{c}$, after 180 days, already reached more than 90% of annual results. Between the 90th and 365th day of curing, a substantial increase of $K_{c}$ was observed only in concrete with 30% LCFA additive, Figure 15.

## 5. Discussion

According to the results, the LCFA used in the study is characterized by high pozzolanic activity (Figure 11). The results thus obtained, allow for the anticipation that the composites made of these additives—sustainable concretes—will be characterized by favorable mechanical parameters, especially after a long period of curing time.

The analysis of fracture processes in these concretes was primarily based on the results of the macroscopic fracture toughness, designated for three modes. This allowed for the estimation of generalized fracture toughness $K_{c}$.

The parameter values of fracture mechanics depend on the age of concretes during the conducted tests. It was observed that the LCFA additive in the amount of 30% of mass of cement reduces the $K_{c}$ after three and seven days after preparing the batch. Due to the high activity of the LCFA (Figure 11), after a month and later, 20% additive of binder substitute guarantees high fracture toughness in matured concretes. In concretes with 30% LCFA additive, its beneficial effect on the indicator of the $K_{c}$ occurs after 180 days.

When comparing the effect of LCFA on the generalized fracture toughness (Figure 14), and compressive strength of concretes (Figure 12), distinct similarities in the effect of used mineral additives on the results obtained in both tests can be observed.

## 6. Conclusions

On the basis of the comprehensive fracture toughness tests, in which a portion of the binder was replaced with active pozzolanic low calcium fly ash, it can be concluded that:

1. It is possible to make sustainable concrete with the addition of low calcium fly ash with high compressive strength and generalized fracture toughness.
2. The low calcium fly ash additive in the amount of 20 and 30% of mass of cement significantly affects the change of generalized fracture toughness in tension, shear and torsion.
3. The values of the critical stress intensity factor $K_{c}$ depend on the age of concrete.
4. The low calcium fly ash additive in the amount of up to 30% of mass of cement drastically reduced the compressive strength and the generalized fracture toughness at an early age.
5. 20% low calcium fly ash additive ensures high compressive strength and generalized fracture toughness in matured concretes.
6. Concretes with 30% low calcium fly ash additive are characterized by the highest dynamic increase of the parameters $f_{cm}$ and $K_{c}$.
7. After 180 and 365 days, generalized fracture toughness of LCFA-30 concrete is higher in comparison to the values obtained for reference concrete.
8. Results of the generalized fracture toughness and the compressive strength are convergent qualitatively.
9. Effect of curing time on the compressive strength and the generalized fracture toughness of concretes with low calcium fly ash additive is directly related to the pozzolanic activity of this ashes.

## 7. Possible Use of the Obtained Results in the Construction Practice

Sustainable concretes with the low calcium fly ash additive are used in building structures, both residential and industrial. Range of composites, made on the basis of the binders modified with this waste is very rich. Such concretes are used in the construction industry: Concrete, reinforced concrete and prestressed; in structures: monolithic, prefabricated, composite and prestressed.

On the basis of the obtained results, several practical tips and recommendations on the use of sustainable concretes, with the low calcium fly ash, in concrete industry can be suggested:

1. In the case of concretes, which are scheduled for commissioning after 28 days, or later, the preferred solution is to use composites modified with the 20% LCFA additive.
2. The use of concretes with 30% LCFA additive in typical structures is possible, however, it should be taken into account that their strength and fracture toughness can be reduced for up to six months, compared to plain concretes.
3. It is not advisable to construct concrete structures, with the low calcium fly ash additive, if their commissioning is planned before the 28th day after the placing of concrete mixture.
4. It is not recommended to use composites with the low calcium fly ash additive in the prefabricated elements, in which the interoperational transport strength would be less than a week.
5. In any case, it is not advisable to use concretes with 20% and 30% low calcium fly ash additive, if they are subjected to any load in less than three days from the preparation of concrete mixture.

## Figures and Tables

**Table 1:** Papers which presented test results of effect of curing time on the parameters of low calcium fly ash (LCFA) composites.

Analyzed Parameter	Curing Age (days)	Reference
Mechanical properties	1, 7, 28, 56, 90, 180, 365	[56]
	3, 14, 28, 49, 91, 182, 364	[57]
	7, 14, 28, 56, 91, 365	[58]
	3, 7, 28, 90, 180	[59]
	3, 7, 14, 28, 90, 180	[60]
Corrosion resistance	Weekly, from 7 to 1000	[61]
Chloride penetration	91	[62]
	28, 180	[63]
Drying shrinkage	3, 7, 28, 56, 180	[63]
Porosity, pore volume distribution and heat development	3, 7, 14, 28, 49, 112, 182, 364	[57]
Water absorbtion and water permeability	28, 90	[59]
Hydration process	1/12, 1/5, 1, 3, 7, 14, 28, 56, 180	[64]
Microstructure	1, 7, 28, 56, 90, 180, 365	[56]
	3, 14, 28, 49, 91, 182, 364	[56]
	1, 7, 28, 56	[64]
	1, 7, 28, 56, 91	[65]
Crack propagation	1, 7, 28, 56, 90, 180, 365	[56]
Fracture properties (in mortar)	7, 28, 90, 180	[66]

**Table 2:** Chemical composition of the LCFA and the OPC.

Chemical	Component (wt %)	
	LCFA	OPC
SiO_2_	50.96	21.37
Al_2_O_3_	25.88	5.02
Fe_2_O_3_	8.25	2.40
CaO	2.15	63.95
Na_2_O	1.26	0.18
K_2_O	2.65	0.91
SO_3_	0.65	3.00
MgO	2.60	2.47
P_2_O_5_	0.35	-
Cs_2_O	0.09	-
BaO	0.32	-
TiO_2_	1.36	-
Cl	-	0.06
LOI ^a^	3.20	1.24

^a^ Loss on ignition.

**Table 3:** Concrete mix design (kg/m^3^).

Concrete Series	OPC	LCFA	%LCFA	Sand	Gravel	Water	Plasticizer
LCFA-00	352	0	0				
LCFA-20	282	70	20	676	1205	141	2
LCFA-30	246	106	30				
